# Rapid Identification of *Asteraceae* Plants with Improved RBF-ANN Classification Models Based on MOS Sensor E-Nose

**DOI:** 10.1155/2014/425341

**Published:** 2014-08-19

**Authors:** Hui-Qin Zou, Shuo Li, Ying-Hua Huang, Yong Liu, Rudolf Bauer, Lian Peng, Ou Tao, Su-Rong Yan, Yong-Hong Yan

**Affiliations:** ^1^Library, Beijing University of Chinese Medicine, No. 11 Bei San Huan Dong Lu, Chaoyang District, Beijing 100029, China; ^2^Beijing University of Posts and Telecommunications, No. 10 Xi Tu Cheng Lu, Haidian District, Beijing 100876, China; ^3^School of Chinese Materia Medica, Beijing University of Chinese Medicine, No. 6 Wang Jing Zhong Huan Nan Lu, Chaoyang District, Beijing 100102, China; ^4^Institute of Pharmaceutical Sciences, University of Graz, Universitaetsplatz 4, 8010 Graz, Austria

## Abstract

Plants from *Asteraceae* family are widely used as herbal medicines and food ingredients, especially in Asian area. Therefore, authentication and quality control of these different *Asteraceae* plants are important for ensuring consumers' safety and efficacy. In recent decades, electronic nose (E-nose) has been studied as an alternative approach. In this paper, we aim to develop a novel discriminative model by improving radial basis function artificial neural network (RBF-ANN) classification model. Feature selection algorithms, including principal component analysis (PCA) and BestFirst + CfsSubsetEval (BC), were applied in the improvement of RBF-ANN models. Results illustrate that in the improved RBF-ANN models with lower dimension data classification accuracies (100%) remained the same as in the original model with higher-dimension data. It is the first time to introduce feature selection methods to get valuable information on how to attribute more relevant MOS sensors; namely, in this case, S1, S3, S4, S6, and S7 show better capability to distinguish these *Asteraceae* plants. This paper also gives insights to further research in this area, for instance, sensor array optimization and performance improvement of classification model.

## 1. Introduction

Chinese herbal medicines are getting more and more international attention based on their alternative treatment for some refractory diseases and chronic disorders. However, it appears in the medicinal materials markets that nowadays some medicines with low quality or even fake materials are sold. This phenomenon results in economic loss, poor clinical effects, and even poisoning. Therefore, the need for efficient and reliable identification and quality control of these herbal medicines is of crucial importance.

In recent years, lots of modern techniques are introduced into traditional Chinese medicine (TCM) analysis, including high performance liquid chromatography (HPLC), mass spectrometry (MS), nuclear magnetic resonance (NMR), and DNA genetic analysis [1]. The whole chemical profile of TCM could be expressed in different fingerprints which are used to identify original materials [2], especially combined with multivariate statistical analyses [3, 4]. As for the analyses of volatile components in TCM, gas chromatography (GC) and gas chromatography-mass spectrography (GC-MS) are the most popular ways to determine volatile components in TCM. However, these methods normally only detect one or more chemical compositions, and most of the given information reflects the fragments instead of the holistic state of the volatile components. Besides, they are time-consuming for complex sample pretreatment and no environmental protection.

Compared to them, metal oxide semiconductor sensors (MOS sensor) electronic nose (E-nose) is a simple, rapid, and noninvasive technology with less sample amount and without organic reagents. The initial and unique chemical form of the volatile components in TCM could be reflected by their response to MOS sensor which can be used to identify different TCM [5, 6]. And the information could be fully collected for further analysis.

E-nose, which has already been applied in various fields in the past decades [7–10], is a very promising method for identifying different samples based on their different information of the responses between their volatile components and the sensors. In these studies, different kinds of data processing methods have been applied to construct the classification models such as probabilistic neural network (PNN) [11], Bayesian neural network (BNN) [12], multilayer perceptrons (MLP) [13], and radial basis function artificial neural network (RBF-ANN). Among them, RBF-ANN shows good performance for classification modeling [14].

Lin et al. [15] employed RBF-ANN to construct a classification model based on E-nose to successfully distinguish different kinds of* Apiaceae* plants. However, there are few studies on the improvement of RBF-ANN classification model combined with the selection and optimization of MOS sensor array. Daqi et al. proposed a type of modular RBF-ANN to improve its performance [16].

In this paper, we aim to develop a novel discriminative model by improving RBF-ANN classification model. Through applying feature selection algorithms, principal component analysis (PCA), and BestFirst + CfsSubsetEval (BC), the construction of networks in RBF-ANN models was simplified, maintaining the same high-quality discriminative ability. Based on feature screening, the redundant information in the original RBF-ANN model for identifying different* Asteraceae* plants was eliminated and more valuable information was retained. Furthermore, using these improved RBF-ANN models, five MOS sensors were selected to possess better capability to distinguish these eight species of* Asteraceae* plants, which are S1, S3, S4, S6, and S7.

## 2. Materials and Methods

### 2.1. Plant Materials

Eight different species of plants, all originating from* Asteraceae* family, were purchased from Beijing Tongrentang Co., Ltd. (Beijing, China) and identified by Professor Yong-Hong Yan in Beijing University of Chinese Medicine (Beijing, China). As shown in Table 1, samples were labeled as* Bai Zhu*,* Cang Zhu*,* Gong Ju*,* Ye Ju Hua*,* Ai Ye*,* Mu Xiang*,* E Bu Shi Cao*, and* Niu Bang Zi*.

### 2.2. E-Nose

E-nose (*α*-FOX3000, Alpha M.O.S., France) consists of 12 MOS sensors, a head space sampler, and a signal processing system. Twelve commercial metal oxide sensors are placed in two rectangular chambers, six per each. A list of all MOS sensors' information and their application is illustrated in Table 2. They are LY2/LG, LY2/G, LY2/AA, LY2/GH, LY2/gCTL, LY2/gCT, T30/1, P10/1, P10/2, P40/1, T70/2, and PA/2, respectively, numbered as S1, S2, S3,…, S12. The sensor response was expressed as the ratio of conductance ((*G* − *G*_0_)/*G*).

Ground into small particles, 0.2 g of each sample was accurately weighed into a 10 mL septa-sealed bottle and loaded into the autosampler tray. After incubation with optimized parameters in the previous research (temperature is 30°C and time is 300 seconds), 2000 *μ*L of headspace air was automatically injected into the E-nose system by a syringe and detected by MOS sensor array. The conductance ratio of each sensor changed during the measurement process. The measurement phase lasted for 120 s, which was enough for all the sensors to reach the stable values and return to the baseline. Signals were collected by the computer and the data acquisition cycle was 1 s.

Six repeated samples were prepared for each kind of plants and totally 48 measurements were performed by the dynamic headspace sampling procedure. The E-nose responses values of those plants were extracted and recorded by the computer. Then different kinds of RBF-ANN models were established to identify them.

### 2.3. Classification Model Improved RBF-ANN Combined with PCA and BC

In the field of mathematical modeling, a radial basis function network is an artificial neural network which uses radial basis functions as activation functions. Normally, it contains three layers: one input layer, one hidden layer (sometimes more than one), and one output layer. The output of the network is a linear combination of radial basis functions of the inputs and neuron parameters. One of the principal problems encountered in the RBF network modeling procedure is that more redundant or uncorrelated information in the input layer may increase the error rate or result in overfitting in the output layer. Hence, in order to solve the above problems, we employ two kinds of feature selection methods, namely, PCA and BC, to process the high-dimension data, eliminating redundant information and selecting the factors which contribute more valuable information to the final target: presenting a rapid and accurate method for identification of these eight species of* Asteraceae* plants.

PCA helps us to figure out which samples are different from the others and which principal components extracted from the original variances contribute more to this difference. Focus on dealing with those principal components with more important information is one way for us to reduce high dimension in data processing.

BC is a kind of feature extraction technologies, which can screen out the characteristic parameter vectors with high relevance to the classification and low relevance to the others. We can get an optimum set of MOS sensors for final identification based on BC.

As for the evaluation of the established models, 10-fold cross-validation method is applied to avoid the overfitting and get the classification accuracy. The classification results should not be considered if the classification accuracy was lower than 80%.

## 3. Results and Discussion

### 3.1. E-Nose Responses to the Volatile Components of Samples from* Asteraceae* Plants

When detecting the sensor response to a given sample, the response values are used as *R* = (*G* − *G*_0_)/*G*, where *R* is the response, *G*_0_ is the conductance of a sensor in the reference air, and *G* is the conductance of the sensor in the sample gas.

**Figure 1:**
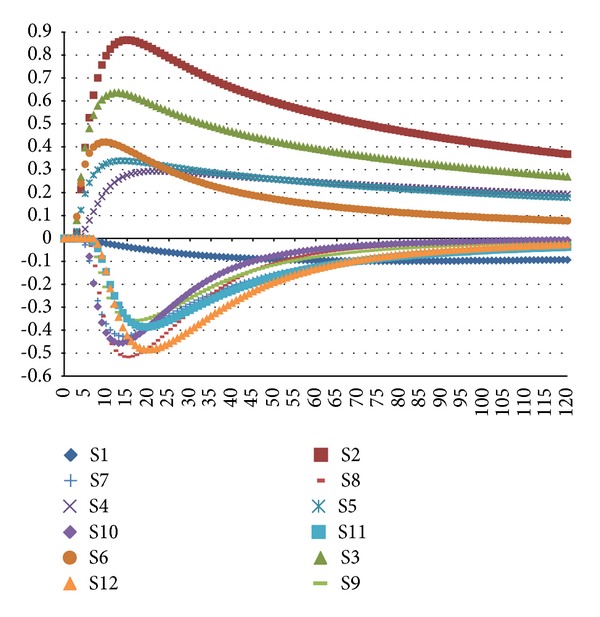
Typical responses of 12 MOSs measuring of a* Cang Zhu* sample.

Figure 1 shows the typical responses of 12 MOS sensors with one sample of* Cang Zhu* (dried Rhizoma of* Atractylodes lancea* (Thunb.) DC.). Each line represents the signals of a* Cang Zhu* sample in one of the 12 MOS sensors. The horizontal axis is the time line, a total of 120 seconds; the vertical axis is the response value of the MOS sensor. The curves represent the resistance value of each sensor against time due to the electrovalve action when the volatile compounds reached the detection chamber. In the initial period, the response value of each sensor was low and then increased continuously and finally stabilized after a few seconds or minutes. In this study, 12 maximum response values of each sample from 12 MOS sensors were extracted and analyzed individually.

The repeatability of the established method was evaluated with six parallel tests of the samples. The relative standard deviation (RSD, *n* = 6) values of 12 MOS sensors were calculated. The results were all less than 3%, proving a high repeatability of E-nose response.

### 3.2. RBF-ANN with Original Data from 12 MOS Sensors

**Figure 2:**
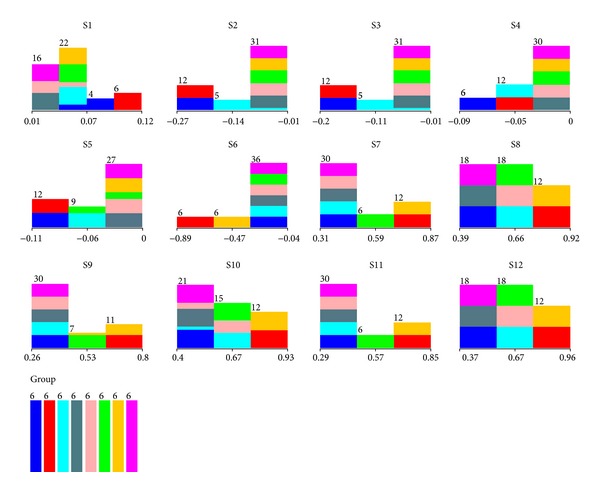
Different contributions of 12 MOS sensors in the original RBF-ANN model for* Asteraceae* plants identification.

Figure 2 shows the different contributions of 12 MOS sensors in the original RBF-ANN model for* Asteraceae* plants. Eight kinds of colors presented eight species of* Asteraceae* plants. Firstly, they are divided into different groups in each sensor. For example, they are four groups in the case of S1 but three groups in the case of S2. That means S1 contributes more valuable information to distinguish these eight species of* Asteraceae* plants into smaller groups. Secondly, the classification situation differs in every sensor. For example, the green samples and the light blue samples are considered as the same group and they could not be separated in the case of S1. However, in the case of S2, S3, S4, S7, S9, and S11, they are separated into different groups. Thirdly, some identification information is overlapped in some sensors. For example, S2 and S3 contain the same information which means, in this identification of* Asteraceae* plants, S2 and S3 have the similar contribution. According to these, it is certain that some of the sensors contain more valuable information for the identification but some of them resemble the others which should be eliminated for model simplification. Therefore, it shows us a potential way to improve the classification model on the basis of sensors screening and optimization.

**Figure 3:**
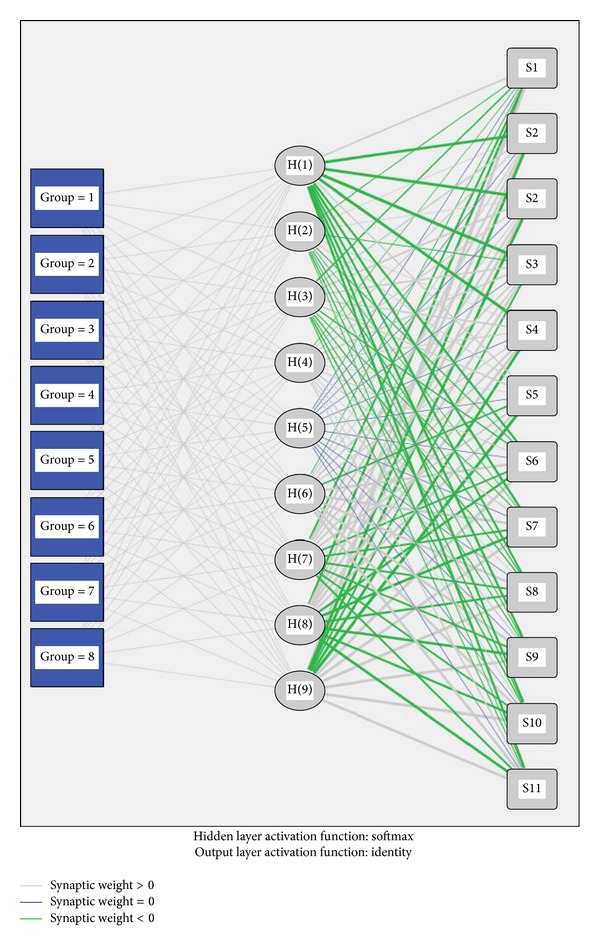
Architecture of three layers of original RBF-ANN with 12 units in the input layer (codes stand for samples and S1~S12 stand for 12 MOS sensors).

Figure 3 shows the architecture of three layers of RBF-ANN for training and identification. The input layer in this network consists of 12 units and the identification result can be gained directly from this model. In this initial RBF-ANN model, input layer contains 12 units from 12 MOS sensors. All the original data of the input layer are imported into the hidden layer and then calculated by the RBF. Afterwards identification results are gained and the samples are divided into eight different groups. Figure 3 tells it is necessary to reduce the high-dimension data in the network so as to simplify the modeling process.

### 3.3. Comparison of Original and Improved RBF-ANN Models

Based on PCA feature selection method, two main factors were selected and the factors with minimum weight were rejected.

Based on BC feature selection method, six MOS sensors (S1, S3, S4, S6, and S7) were screened out to contribute the most valuable information to identify these eight species of* Asteraceae* plants.  Table 3 shows that the classification accuracies of three types of RBF-ANN models with 12, 2, and 5 units by 10-fold cross-validation are all 100%. That means the RBF-ANN still can achieve the identification goal by lower dimension data reduced by these two kinds of feature selection methods. Meanwhile, the sum of square error is decreased in the improved RBF-ANN models combined with PCA as well as with BC. Last but not least, it is suggested BC should be considered as a method for optimizing the set of sensor array. Further research on which type of sensor is more sensitive and exclusive to volatile components in TCM should be carried on.

## 4. Conclusions

Lots of plants originating from the* Asteraceae* family are applied as Chinese herbs and beverage ingredients in Asian areas, particularly in China. However, they may be confused due to their similar odor, especially when they are ground into powder, losing the typical macroscopic characteristics. In this paper, E-nose was employed to extract and analyze the volatile components fingerprints of eight species of* Asteraceae* plants. Then RBF-ANN was applied to establish the classification model. Furthermore, two different kinds of feature selection methods, PCA and BC, were used to solve high-dimension data problem. Through PCA and BC, we have synthesized numerous criteria, eliminated information overlapping of the sample, and reduced the input dimensions of RBF network. And it is the first time to introduce feature selection methods to improve RBF-ANN classification model and get valuable information on how to attribute more relevant MOS sensors. In this paper, S1, S3, S4, S6, and S7 show better capability to distinguish these eight species of* Asteraceae* plants.

In a word, this paper presents a rapid, accurate, and effective method to distinguish* Asteraceae* plants. Also it gives insights into further studies, for instance, to search some kinds of unique sensors which are more sensitive and exclusive to volatile components in TCM, to improve the identification ability of E-nose. Besides, screening sensors made by other novel materials would be also an interesting way to improve identification capability of E-nose [17].

## Figures and Tables

**Table 1:** *Asteraceae* plant as an herbal medicine.

Number	Label	Herbal name
1	*Bai Zhu *	Dried Rhizoma of *Atractylodes macrocephala* Koidz.
2	*Cang Zhu *	Dried Rhizoma of *Atractylodes lancea* (Thunb.) DC.
3	*Gong Ju *	Dried Flos of *Chrysanthemum morifolium* Ramat.
4	*Ye Ju Hua *	Dried Flos of *Chrysanthemum indicum* L.
5	*Ai Ye *	Dried Folium of *Artemisia argyi* Levl. et Vant.
6	*Mu Xiang *	Dried Radix of *Aucklandia lappa* Decne.
7	*E Bu Shi Cao *	Dried Herba of *Centipeda minima* (L.) A. Br. et Aschers.
8	*Niu Bang Zi *	Dried Fructus of *Arctium lappa* L.

**Table 2:** Main application of 12 MOS sensors in *α*-FOX3000 E-nose.

Number	Name	Main application
S1	LY2/LG	Oxidizing gas
S2	LY2/G	Ammonia/carbon monoxide
S3	LY2/AA	Ethanol
S4	LY2/GH	Ammonia/organic amine
S5	LY2/gCTL	Hydrogen sulfide
S6	LY2/gCT	Propane/butane
S7	T30/1	Organic solvents
S8	P10/1	Hydrocarbons
S9	P10/2	Methane
S10	P40/1	Fluorine
S11	T70/2	Aromatic compounds
S12	PA/2	Ethanol/ammonia/organic amine

**Table 3:** Comparison of three types of RBF-ANN with 12, 2, and 5 units.

Sum of square error	Training	Testing	Classification accuracy
			via 10-fold cross-validation
12 units RBF-ANN	0.939	0.320^a^	100%
2 units RBF-ANN	0.083	0.029^a^	100%
5 units RBF-ANN	0.522	0.207^a^	100%

## Abbreviations

TCM: Traditional Chinese medicine
E-nose: Electronic nose
MOS: Metal oxide semiconductor
PCA: Principal component analysis
BC: BestFirst + CfsSubsetEval
RBF-ANN: Radial basis function artificial neural networks.
